# The Hot Springs Hospital

**Published:** 1887-04

**Authors:** 


THE HOT SPRINGS HOSPITAL.

Several years ago the late Senator Logan visited Hot Springs, Arkansas, and obtained there so much relief from rheumatic troubles with which he was afflicted that he determined to make the benefits of the place available for the army and navy, and to that end obtained an appropriation for the construction of a hospital at Hot Springs for the use of the officers and men of those services. The sketch plans for this hospital were prepared by Dr. Billings, of the army, and these, after approval by the Secretary of War, were elaborated, and detailed drawings and specifications prepared by a Washington architect, Mr. Smithmeyer. The buildings are now completed, Dr. R. S. Vickery, of the army, has been assigned as surgeon in charge, and a circular from the War Department announces that the hospital will be opened for the reception of patients January 17th, 1887. The circular says:

" Relief may reasonably be expected from the use of the Hot Springs water in the following classes of diseases, viz.: gout and rheumatism in their various forms, after the acute or inflammatory stage has passed; neuralgia, peripheral or central, especially when depending upon gout, rheumatism, specific infection, or metallic poisoning; paralysis, if not recent, progressive or organic; locomotor ataxia, or tabes, if not in advanced stages, and especially if traceable to specific infection; Bright's disease of the kidneys, only in the early stages; diseases of the bladder and urinary organs; functional diseases of the liver; dyspepsia; chronic diarrhœa and catarrhal diseases generally; chronic skin diseases, especially of the squamous or scaly forms; chronic conditions, resulting immediately from specific infection, either syphilitic or malarial; chronic alcoholism.

"In general terms it may be stated that the Hot Springs water acts by stimulating all secretions and organic functions, increasing appetite, promoting digestion and assimilation; favoring tissue change and excretion of waste products, relieving internal congestions, and stimulating the blood-making function.

"In the following classes of diseases the use of the Hot Springs water is contra-indicated: All acute inflammatory diseases; tuberculosis; organic diseases of the heart and brain; aneurism, cancer and all diseases in which stimulation of the circulation is to be avoided."

The hospital will accommodate about twenty officers and sixty-four enlisted men. Authority for admission to it is to be obtained from the Adjutant General of the army or from the Surgeon-General of the navy.

In Europe it is very common to provide special hospital accommodation at certain mineral or thermal springs for the benefit of the army, but this is the first time it has been tried in this country, and the results will be looked for with interest. It should be possible from the records of this hospital to obtain much more definite information than we at present possess as to the real thereapeutic efficacy of the Hot Springs waters.—*Medical News.*

